# Dose-Dependent Relationship between Protection of Thioacetamide-Induced Acute Liver Injury and Hyperammonemia and Concentration of Lactobacillus salivarius Li01 in Mice

**DOI:** 10.1128/spectrum.01847-21

**Published:** 2021-12-22

**Authors:** Pengcheng Lou, Yangfan Shen, Aoxiang Zhuge, Longxian Lv, Xueling Zhu, Yin Yuan, Liya Yang, Kaicen Wang, Bo Li, Lanjuan Li

**Affiliations:** a State Key Laboratory for Diagnosis and Treatment of Infectious Diseases, National Clinical Research Center for Infectious Diseases, Collaborative Innovation Center for Diagnosis and Treatment of Infectious Diseases, The First Affiliated Hospital, Zhejiang University School of Medicine, Hangzhou, China; b Research Units of Infectious Disease and Microecology, Chinese Academy of Medical Sciences, Beijing, China; Emory University School of Medicine

**Keywords:** *Lactobacillus salivarius*, probiotic, acute liver failure, hyperammonemia, thioacetamide

## Abstract

Recently, probiotics have been widely used as an adjuvant therapy to cure, prevent, or improve certain diseases. However, no research has been carried out into the dose of probiotics, especially the maximum dose. Therefore, the effective and safe dosage of probiotics needs to be studied. Recently, L. Yang, X. Bian, W. Wu, L. Lv, et al. (Microb Biotechnol 13:1860–1876, 2020, https://doi.org/10.1111/1751-7915.13629) discovered that Lactobacillus salivarius Li01 had a protective effect on thioacetamide-induced acute liver injury and hyperammonemia, and a fixed concentration (3 × 10^9^ CFU/mL) of *L. salivarius* Li01 was applied in their study. However, the most effective treatment concentration of *L. salivarius* Li01 remains unknown. Therefore, four concentration gradients of *L. salivarius* Li01 suspension were prepared for groups of mice to have different levels of bacterial colonization by gavage. Then, acute liver injury and hyperammonemia were induced via thioacetamide administration. By observation and detection, an inverted U-shaped protective effect from *L. salivarius* Li01 existed in thioacetamide-induced acute liver injury and hyperammonemia. Of note, significant deterioration was confirmed within the group that was orally administered with an excessive concentration of *L. salivarius* Li01 suspension, and this was attributed to endotoxemia that resulted from compromised immunity, a damaged intestinal barrier, and bacterial translocation.

**IMPORTANCE** This research investigated the relationship between the concentration of Lactobacillus salivarius Li01 and its impact on mice that had a thioacetamide-induced acute liver injury and hyperammonemia. These findings could provide new insights into the effective, proper, and safe use of probiotics.

## INTRODUCTION

Recently, combined with progressive research into gut microbiota, the function of the gut-liver axis and gut-brain axis has started to play an important role in many different diseases (1, 2). In addition, the number of studies and clinical practices involving modulating the gut microbiota is increasing in an attempt to prevent, ameliorate, or discover a cure for some diseases. This could help to determine new treatments for some intractable diseases. Among these new practices, viable and successful ones remain limited, with probiotics and fecal microbiota transplantation (FMT) being the most common germ therapies to date (3). According to the relevant literature, the two most common indications for FMT are Clostridium difficile infection and antibiotic-associated diarrhea (4). Recently, FMT application was tentatively extended to other diseases, such as irritable bowel diseases (5), obesity (6), and diabetes (7). Since the FMT procedure entails several steps, which include culture collection, donor screening, and enteral tubing, its standardization, safety, and effectiveness require further study (8, 9). In addition, enteral tubing is an invasive operation; therefore, it is not commonly used due to its inconvenience. And for probiotics, due to their long history of application in human beings (10) and oral administration, they are considered safer and more convenient than FMT and, therefore, are applied more often.

From the traditional *Lactobacillus* sp. and *Bifidobacteria* sp. to the current *Saccharomyces boulardii* (11) and an engineered Escherichia coli (Nissle) (12), these probiotics have been defined as live microorganisms that when administered in adequate amounts confer a health benefit to the host (13). Currently, there are no officially approved treatments by probiotics worldwide; however, an increasing number of studies have proved that different probiotics and their combinations have a profound influence on many diseases. In most studies, the concentrations of probiotics ranged from 10^8^ to 10^10^ CFU/mL (14–16); however, no specific experiments exist on the upper limit of the dose of probiotics. Therefore, further studies are required to address this issue.

Liver disease is a field in which the role and mechanism of probiotics have been studied extensively. Because of the gut-liver axis, there are bidirectional relationships between the gut combined with its microbiota and the liver. The gut-liver axis forms as a result of the interactions between signals that are generated by dietary, genetic, and environmental factors. Given the interdependent relationship between the gut and the liver, it is easy to understand why dysbiosis in the gut might cause or worsen a range of hepatic diseases. Changes in the liver could reshape the gut microbiota (17). Based on the features of the gut-liver axis, in which the gut mucosal barrier and microbiota play pivotal roles, probiotics could exert a significant influence on the gut-liver axis, which includes immunomodulatory and anti-inflammatory effects on the intestinal microflora and intestinal barrier functions (18). Based on this, probiotics have been used to treat a variety of liver diseases (19–22).

Among liver diseases, acute liver failure (ALF), which is a dangerous disease with a mortality rate of ≤30%, along with its complication hepatic encephalopathy (HE), poses a significant threat to human health and places a large burden on health care services (23, 24). ALF is a clinical syndrome with a poor prognosis, and its causes vary from acetaminophen toxicity, liver ischemia, and autoimmune hepatitis to viral hepatitis and drug-induced liver injury (24). Before broad access to liver transplantation, the mortality rate for ALF was 80%. Then, with the development of liver transplantation and the help of supportive treatments in the intensive care unit (ICU), the mortality decreased to approximately 33% (25). However, due to a scarcity of donors’ livers, liver transplantation cannot meet the needs of all ALF patients. Therefore, more research needs to be carried out to address the challenge brought by ALF.

Despite different pathogenesis of acute liver injuries, their clinical characteristics resemble those found in reference 24, which allows us to establish various ALF models to study the influence of ALF on the body and its mechanism. The most typical animal models for ALF with HE, namely, the type-A HE, were discovered to be the hepatic devascularized model and the animal model with thioacetamide (TAA)-induced toxic liver injury (26). TAA is a typical liver toxin, and it has been widely used to establish liver injury model for decades. Severe hepatocellular and bridging necrosis without cholestasis was observed in rats and mice with TAA treatment (27). In addition, the neurobehavioral abnormalities after TAA injection were similar to those of HE (28).

Recently, Yang et al. (29) found that in the mouse model with thioacetamide-induced acute liver injury and hyperammonemia, *L. salivarius* Li01 had a protective effect, therefore improving the survival rate of the mice. Apart from that, *L. salivarius* Li01, which is isolated from the feces of healthy individuals, has also been proved to have bile salt resistance (30), ameliorates acute liver injury induced by d-galactosamine (31), and prevents CCl_4_-induced cirrhosis by protecting the intestinal barrier in rats (32). However, all these studies used fixed concentrations of *L. salivarius* Li01 to test its efficacy on relevant animal models. Therefore, the most effective concentration of *L. salivarius* Li01 remains unknown.

Therefore, based on the research of Yang et al. (29), this study investigated the protective effect that was produced by *L. salivarius* Li01 at different concentrations in mice with TAA-induced acute liver injury and hyperammonemia.

## RESULTS

### Effect of *L. salivarius* Li01 on the weight change and survival rate of mice after TAA administration.

**FIG 1:**
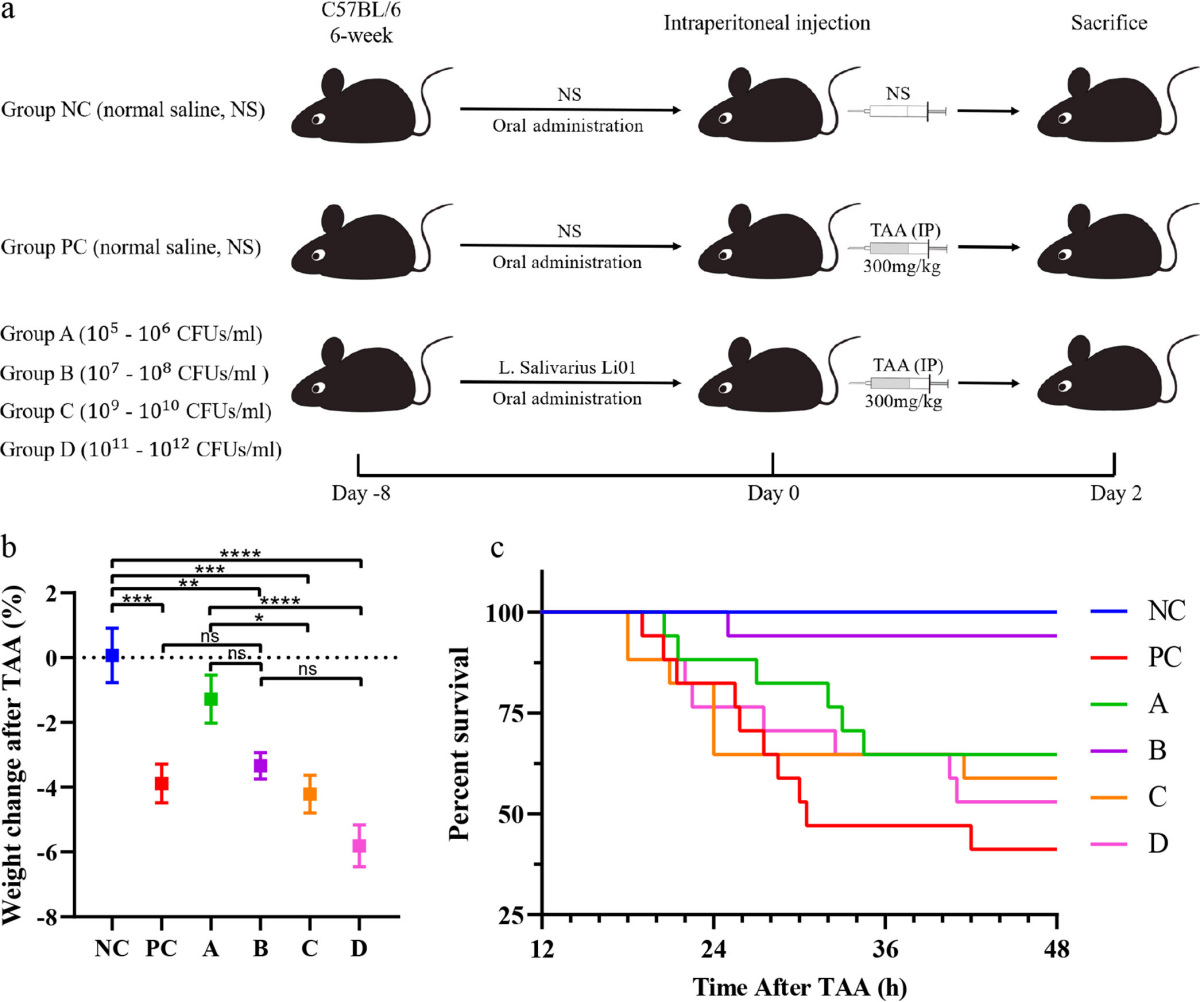
Effect of *L. salivarius* Li01 on the survival rate and weight change in mice after TAA administration. (a) Experimental process diagram. Concentrations in group A, 10^5^ to10^6^ CFU/mL, group B, 10^7^ to 10^8^ CFU/mL, group C, 10^9^ to 10^10^ CFU/mL, group D, 10^11^ to 10^12^ CFU/mL. (b) Percent weight change in mice after TAA administration. (c) Kaplan–Meier survival curves; death numbers in different groups were checked every half hour. Data are presented as mean ± standard error of the mean (SEM). *, *P < *0.05; **, *P < *0.01; ***, *P < *0.001; ****, *P < *0.0001; ns, no significance.

In this study, the mice were divided into six groups; mice in groups NC (negative control) and PC were given normal saline, while groups A, B, C, and D were given 0.3 mL of *L. salivarius* Li01 suspensions by gavage at a fixed time every day on eight consecutive days for four concentration ranges (10^5^ to 10^6^, 10^7^ to 10^8^, 10^9^ to 10^10^, and 10^11^ to 10^12^ CFU/mL; the according absolute dosages corresponding to these concentration ranges are 3 × 10^4^ to 3 × 10^5^, 3 × 10^6^ to 3 × 10^7^, 3 × 10^8^ to 3 × 10^9^, and 3 × 10^10^ to 3 × 10^11^ CFU, respectively). After that, all groups except for group NC were given a single intraperitoneal administration of 300 mg/kg TAA, and group NC was injected with the corresponding volume of normal saline (Fig. 1a).

The bodyweights of the mice in the experiment were measured at two separate time points, one before TAA administration and the other 24 h after TAA administration, and then their weight change percentage was calculated (Fig. 1b). Calculation formula: (weight 24 h after TAA administration − weight before TAA administration)/weight before TAA administration × 100%. 24 h after TAA administration. Groups A and B had a weight change lower than that of the positive control (PC) and groups C and D.

Forty-eight hours after TAA administration, the survival rates of different groups were as follows: NC (13/13), PC (7/17), A (11/17), B (16/17), C (10/17), and D (9/17) (Fig. 1c). Group B had the highest survival rate compared with other groups that received a TAA dose (log-rank test, *P < *0.005).

### Effect of *L. salivarius* Li01 on liver injuries.

**FIG 2:**
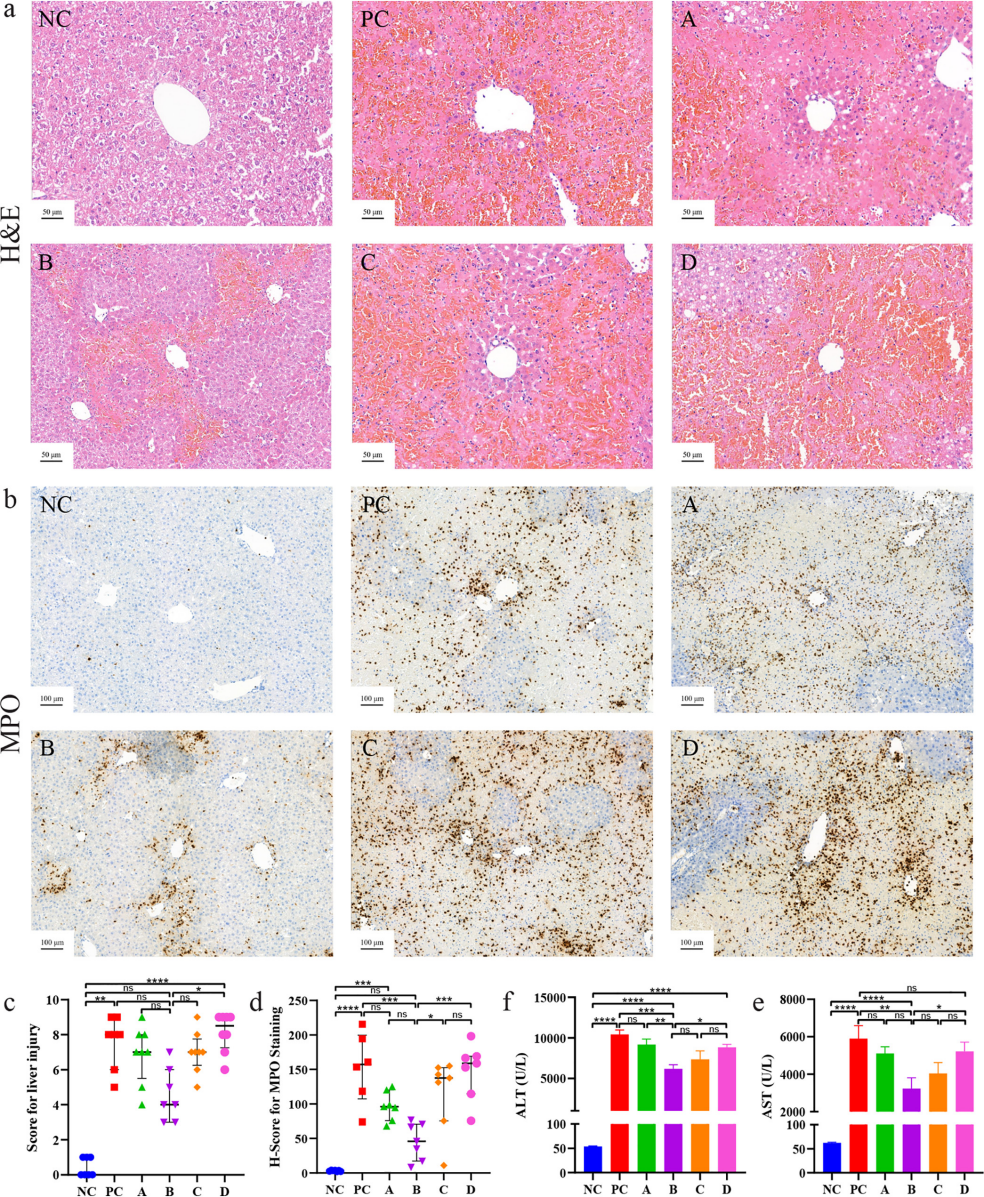
Effect of *L. salivarius* Li01 on liver injuries. (a) H&E staining. (b) Immunohistochemistry of MPO. (c) Scoring of the liver histopathology. (d) H-score for MPO staining. Serum levels of (e) ALT and (f) AST. Data are presented as median with interquartile range in panel c and mean ± SEM in panels d, e, and f. *, *P < *0.05; **, *P < *0.01; ***, *P < *0.001; ****, *P < *0.0001; ns, no significance.

From the liver hematoxylin and eosin (H&E) staining result, the mice in each group that received TAA had different degrees of liver injury (Fig. 2a). These injuries were assessed from the number of cells with degeneration and necrosis, increase in eosinophils, and several inflammatory cells infiltrated around the portal area. Between the groups that received TAA, group B had a mild liver injury, followed by group A, and in groups C, D, and PC, the damage was more severe (Fig. 2a). Scoring of H&E staining was completed according to previous studies (33, 34). From the scoring results (Fig. 2c), group B had a lower score than the other groups except for NC.

Myeloperoxidase (MPO) is a hemoprotein which is expressed mainly in neutrophils and slightly in monocytes and some types of macrophages. This well-known enzyme is released into extracellular fluid during inflammation and catalyzes the conversion of chloride ions (CL^−^) and hydrogen peroxide (H_2_O_2_) into hypochlorite (ClO^−^). ClO^−^ is a highly toxic oxidant which can react with a variety of cell components (35, 36). It could be preliminarily judged that there were different degrees of inflammation around the portal area; however, the aggregation of inflammatory cells could not be observed clearly. Therefore, to clarify the severity of the inflammation in the liver, MPO immunohistochemistry staining was carried out to observe and evaluate the density of inflammatory cells in liver tissues around the portal areas. From the result of MPO staining (Fig. 2b), neutrophil aggregation in the hepatic periportal areas in group B was slighter than that in groups PC, A, C, and D. In addition, the neutrophil aggregation in groups C and D was more obvious than that in group PC, which indicated a more serious inflammatory response and degree of liver injury. In addition, histochemical scores (H-scores) for MPO immunohistochemistry staining were calculated by computer to quantitively assess the inflammation level. The results (Fig. 2d) are in parallel with those of H&E staining and blood biochemical indicators.

Serum alanine aminotransferase (ALT) and aspartate aminotransferase (AST) are considered the main biomarkers of liver injury. The levels of ALT and AST in group B (intermediate concentration) were significantly lower than those in groups PC and D (Fig. 2e and f), which agreed with the histological results.

### Effect of *L. salivarius* Li01 on mitigation of the TAA‐induced inflammatory response.

**FIG 3:**
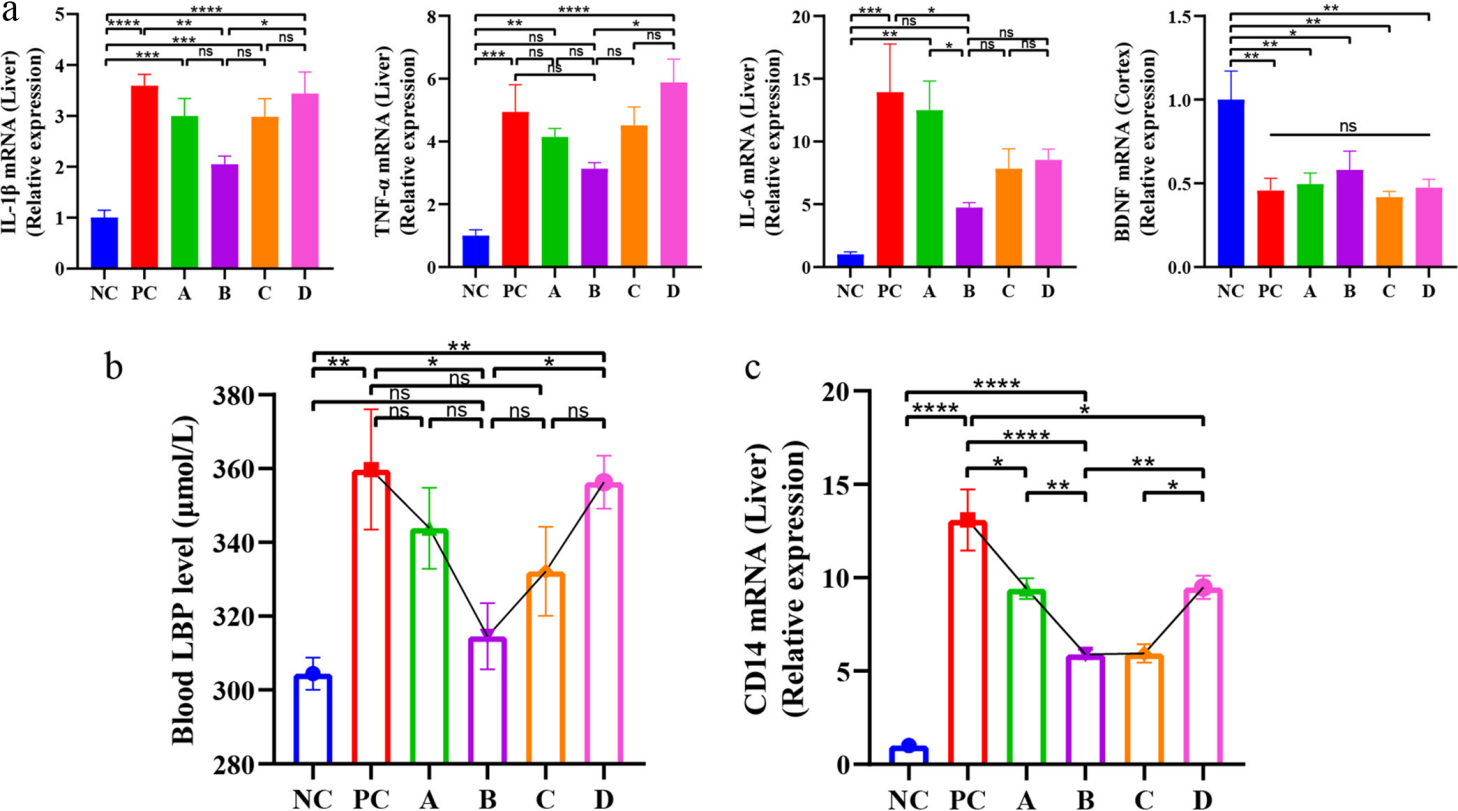
Effect of *L. salivarius* Li01 on the mitigation of TAA-induced inflammatory response. (a) Relative mRNA expression of IL-6, IL-1β, and TNF-α in liver tissue and BDNF in cortex tissue. (b) Blood LBP level in each group. (c) Relative mRNA expression of CD14 in the liver. Data are presented as mean ± SEM. *, *P < *0.05; **, *P < *0.01; ***, *P < *0.001; ****, *P < *0.0001; ns, no significance.

In patients with cirrhosis and HE, proinflammatory cytokines, such as tumor necrosis factor α (TNF-α), interleukin 1β (IL-1β), and interleukin 6 (IL-6), modulate and have synergistic effects with ammonia during cognitive decline (37). In the study by Amanzada et al. (38), thioacetamide induced chemokines and cytokines before neutrophils and macrophage recruitment. Therefore, the mRNA expression levels of the three proinflammatory cytokines (TNF-α, IL-1β, and IL-6) in the liver and one neurotrophin (brain-derived neurotrophic factor [BDNF]) in the brain cortex were investigated. In the liver, the mRNA expression of the three cytokines had an inverted U-shaped phenomenon (Fig. 3a). In group B, the mRNA expression of the three cytokines was the lowest, followed by that in groups A and C, and finally, that in group PC was the highest. The amount of mRNA expression varies; however, the trend was consistent. And for BDNF in the cortex, there was no significant difference between the model groups (Fig. 3a), which meant that the damage in the brain might not have the same trend as that in the liver.

### Effect of *L. salivarius* Li01 on the reduction in serum lipopolysaccharide-binding protein levels.

When the intestinal barrier is compromised, the bacteria in the lumen and their metabolites break through the barrier and enter the body, which results in endotoxemia. Lipopolysaccharide (LPS) is the main component of the outer membrane of Gram-negative bacteria; it binds to the toll-like receptor 4 (TLR4)–MD2 complex and activates the innate immune response. Lipopolysaccharide-binding protein (LBP) and CD14 catalyze the transfer of LPS to TLR4–MD2, in which CD14 and MyD88, the coreceptors of TLR4, activate the NF-κB pathway (39). Therefore, by detecting serum LBP (Fig. 3b) (*P < *0.05), we determined the blood endotoxin level in each group. The result reflected that group B had the minimum level of serum LBP, and compared with groups A and D, there were significant differences (Fig. 3b). In addition, the relative mRNA expression of CD14 in the liver was detected, which serves as a TLR-4 synergist, and the overall trend was consistent with the level of blood LBP (Fig. 3c). The expression of CD14 in groups B and C was comparatively lower than that in groups A and D.

### Effect of *L. salivarius* Li01 on maintaining the integrity of the intestinal barrier.

**FIG 4:**
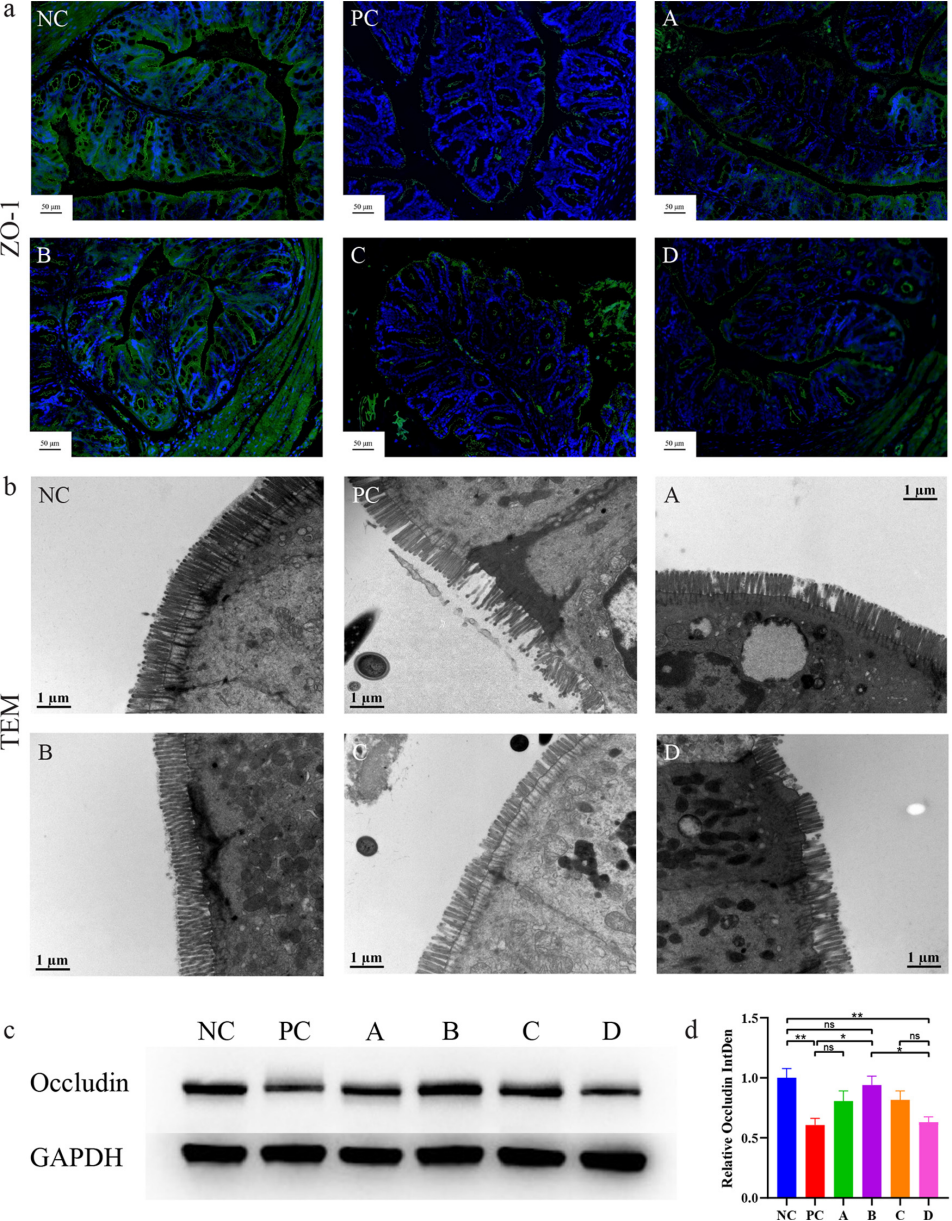
Effect of *L. salivarius* Li01 on maintaining the integrity of the intestinal barrier. (a) Immunofluorescence staining of ZO-1. (b) TEM images. (c) Representative Western blotting results. (d) Quantifications of Occludin expression. Data are presented as mean ± SEM. *, *P < *0.05; **, *P < *0.01; ns, no significance.

The changes in the intestinal bacterial structure are related to the immune response and intestinal barrier integrity (40). Therefore, immunofluorescence staining of zonula occludens‐1 (ZO-1) protein was carried out on the intestinal tissue of mice. ZO-1 is one of the tight junction proteins that can influence transepithelial permeability that includes the intestinal intercellular junctions (41, 42). From the results of the immunofluorescence staining (Fig. 4a), enrichment of ZO-1 in the intestinal epithelium was observed in group B and the other groups, and the fluorescence was fainter, especially in groups PC and D.

However, when immunofluorescence staining was observed under a light microscope, the ultrastructure of the intestine remained invisible. Therefore, the colon tissue specimens were processed further and their fine structures, such as intestinal epithelial cells and microvilli, were observed using transmission electron microscopy (TEM). The TEM result showed that in group B, the intestinal microvilli were more dense and complete, followed by groups A and C, and in groups PC and D, there were obvious defects in the microvilli (Fig. 4b).

To further determine the difference, we performed Western blotting for protein quantification using another tight junction protein, Occludin (Fig. 4c). The relative expression of Occludin in each group is consistent with our observation in ZO-1 immunofluorescence staining and TEM results (Fig. 4d).

### Effect of *L. salivarius* Li01 on ammonia levels in blood and intestinal contents.

**FIG 5:**
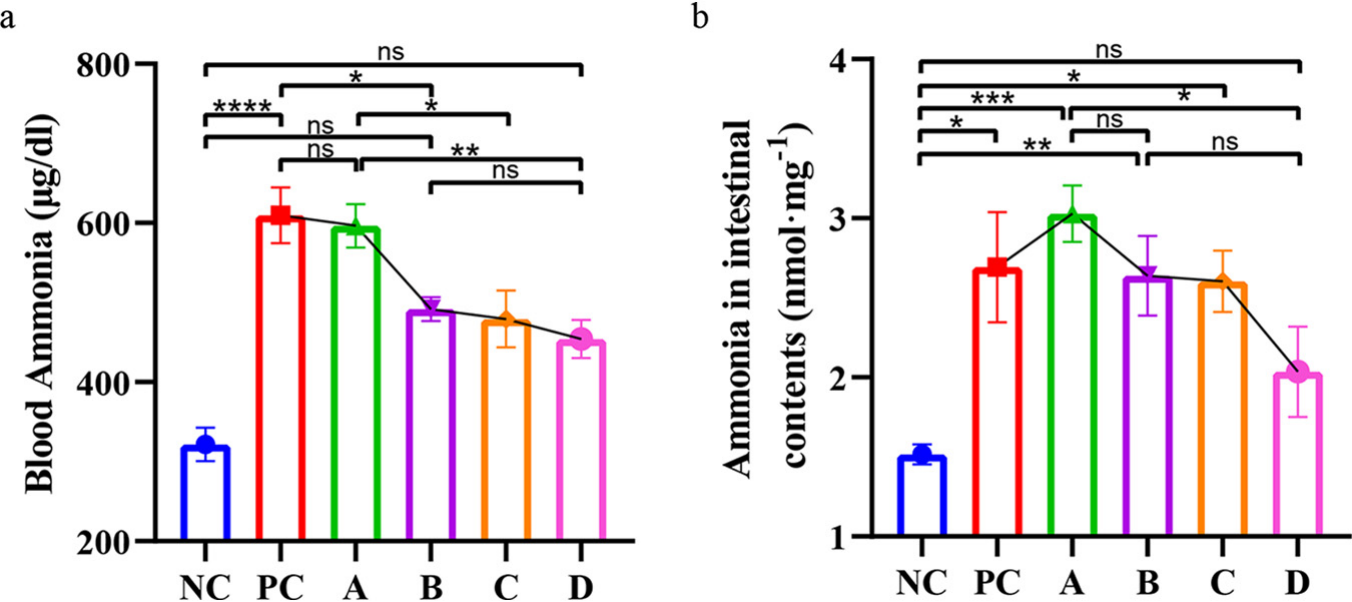
Concentration-dependent effect on ammonia levels in blood and intestinal contents. (a) Blood ammonia levels. (b) Ammonia concentration in intestinal contents. Data are presented as mean ± SEM. *, *P < *0.05; **, *P < *0.01; ***, *P < *0.001; ****, *P < *0.0001; ns, no significance.

Ammonia plays an important role in ALF and HE. Therefore, ammonia levels were detected in the intestinal contents and blood of mice. The results were different from the results from other experiments. Between groups A, B, C, and D, the basic trend was that the ammonia levels in the blood (Fig. 5a) and intestinal contents (Fig. 5b) decreased with increasing concentrations of *L. salivarius* Li01.

### Different concentrations of *L. salivarius* Li01 lead to different structural changes in gut microbiota.

To investigate the effect of different concentrations of *L. salivarius* Li01 on the gut microbiota in mice, the 16S rRNA gene from feces collected 24 h after TAA administration was sequenced. Changes in the structure of gut microbiota were characterized by sequencing bacterial 16S rRNA V3–V4 regions of the fecal samples. A total of 55,201 valid tags were filtered, and 2,472 qualified operational taxonomic units (OTUs) were clustered based on ≥97% sequence identity.

**FIG 6:**
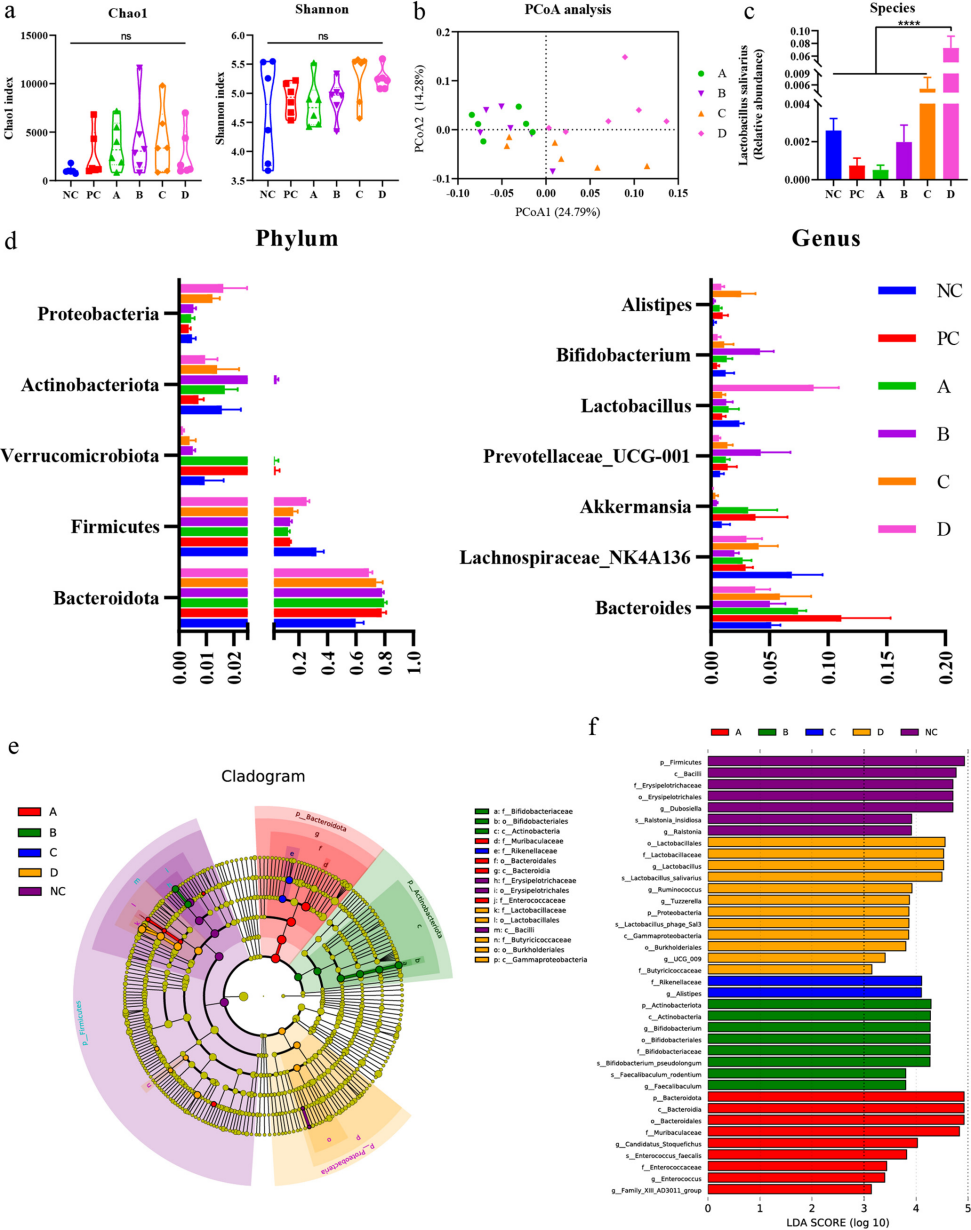
Alterations in gut microbiota after TAA injection. (a) Chao1 index and Shannon index in each group. (b) PCoA analysis of groups A, B, C, and D. (c) Relative abundance of *L. salivarius* (*P < *0.0001). (d) Relative abundance of the most abundant taxa at phylum and genus levels. (e) LEfSe cladogram. (f) Discriminative biomarkers with an LDA score of >3.0.

The α-diversity of the gut microbiota was assessed using the Chao1 and Shannon indices, and both showed no significant differences between the groups (Fig. 6a), which indicated that the overall microbial diversity, richness, and evenness were similar between the groups. The β-diversity was calculated by analysis of similarities (Anosim) and principal coordinates analysis (PCoA), and PCoA was based on weighted UniFrac metrics, which, respectively, explained 24.79% and 14.28% of the variance with PC1 and PC2. Anosim showed that there were significant differences between group D and other groups, groups A and C, groups B and C, and especially between groups B and D, with a large disparity (*P = *0.001) (Table S1). PCoA revealed a distinct separation of group D from groups A, B, and C and a partial separation of group C from groups A and B (Fig. 6b), which agreed with the result from Anosim.

To investigate further the microbial disparity between different groups, the relative abundance of *L. salivarius* was compared at the species level and the relative abundance of the most abundant taxa was compared at phylum and genus levels. The relative abundance at the species level of *L. salivarius* was higher in group D, with a significant disparity with groups A, B, and C (Fig. 6c). The increasing trend of the relative abundance combined with the concentration of *L. salivarius* proved the success of the oral administration. For the taxa at phylum and genus levels, the results indicated that at the phylum level (Fig. 6d, left), there were significant differences in *Bacteroidota* between groups NC and PC, NC and A, and NC and B (*P < *0.01) and no significant differences between the higher-concentration groups (groups C and D) and lower-concentration groups (groups A and B). However, for *Firmicutes*, significant differences existed between group D and groups A, B, and PC, respectively (*P < *0.05), of which group D was more abundant. Apart from that, group B was abundant in *Actinobacteriota*, especially compared with groups C and D (*P < *0.05). At genus level (Fig. 6d, right), differences were noted between group B and groups C and D for *Bifidobacterium* (*P < *0.05), of which group B had a higher abundance.

Finally, to determine the key bacteria associated with this experiment in different groups, we performed linear discriminant analysis effect size (LEfSe) analysis (Fig. 6e and f). This showed that group NC had more abundant taxa than any other groups, group PC had no distinct taxa, and groups A and C had fewer abundant taxa (Fig. 6e). In group D, the discriminative biomarkers consisted of branches associated with *L. salivarius* that were due to the excessive concentration administered and *Butyricicoccaceae* at the family level, *Burkholderiales* at the order level, and *Gammaproteobacteria* at the class level. Group B had a higher abundance that ranged from *Bifidobacteriaceae* at the family level and *Bifidobacteriales* at the order level to *Actinobacteria* at the class level. For group A, the abundant taxa were *Muribaculaceae* at the family level, *Bacteroidales* at the order level, and *Enterococcaceae* at the family level.

## DISCUSSION

Probiotics have been used as a treatment modality for over 100 years. Multitude of studies have been carried out to understand the function of probiotics. However, very few studies have been conducted that examine the dose-response relation. Therefore, based on the research of Yang et al. (29), the protective effect of different concentrations of *L. salivarius* Li01 on TAA-induced acute liver injury and hyperammonemia was explored in this study.

We found the overall trend of ammonia levels in intestinal contents and blood to decrease with the increase of intervention concentration of Lactobacillus salivarius Li01. Yang et al. proved that *L. salivarius* Li01 reduced fecal ammonia, and a correlation has been established between the concentrations of blood ammonia, fecal ammonia, and fecal microbiota (29). This study demonstrated that pretreatment of mice with different concentrations of *L. salivarius* Li01 leads to different amounts of *L. salivarius* Li01 colonizing their lumen, changes the composition and function of intestinal microbial structure, and therefore, affects their intestinal ammonia metabolism. Finally, a concentration-dependent effect of ammonia on their intestinal contents was observed. Accordingly, due to the different levels of gut-derived ammonia in different groups, the ammonia that entered the body via the portal vein was different. Likewise, after having been partly metabolized in the liver, the blood ammonia in different groups also appeared to have a concentration-dependent phenomenon.

However, in accordance with the research findings of Yang et al., as well as the hypothesis of ammonia intoxication (2), lower intestinal ammonia production and lower blood ammonia levels should have yielded a better prognosis. The lower the amount of ammonia that passes through the blood-brain barrier, the lower the probability of brain inflammation and brain edema, and consequently the lower the mortality rates. However, the lower survival rate in groups C and D did not agree with this hypothesis. With lower ammonia levels in both the intestinal contents and the blood, groups C and D should have had higher survival rates. What could have accounted for such difference?

Our results demonstrated that in addition to suffering severe organ injury, group PC (without *L. salivarius* Li01 intervention) had the lowest survival rate. The intervention with *L. salivarius* Li01 in group A (10^5^ to 10^6^ CFU/mL, 3 × 10^4^ to 3 × 10^5^ CFU) and group B (10^7^ to 10^8^ CFU/mL, 3 × 10^6^ to 3 × 10^7^ CFU) improved the acute liver injury and hyperammonemia that were induced by TAA. The general condition of mice improved with increase in probiotic concentration. However, for group C (10^9^ to 10^10^ CFU/mL, 3 × 10^8^ to 3 × 10^9^ CFU) and group D (10^11^ to 10^12^ CFU/mL, 3 × 10^10^ to 3 × 10^11^ CFU), the results exhibited the opposite effect, with the improvement exerted by *L. salivarius* Li01 decreasing and starting to deteriorate with increased concentration. The overall trend was an inverted U shape, and this trend was reflected in many experimental results, such as the survival rate, change in body weight, liver injury, intestinal barrier damage, and liver cytokines.

Different doses of *L. salivarius* Li01 caused changes in the structure of the intestinal flora and metabolites, which was reflected in a dose-dependent inverted U-type protective pattern. Therefore, to determine if this effect could be linked, the blood LBP levels in mice were measured to verify our results. LPS, or endotoxin, is a unique cell wall component of Gram-negative bacteria. It is not easy for LPS to reach other organs or systems in human body through the intestinal barrier under normal conditions. However, with compromised immune system and damaged intestinal barrier, intestinal flora, dominated by Gram-negative bacteria and their LPS, break through the defenses, which causes bacterial translocation and leads to serious complications, such as various infections, endotoxemia, and in severe cases, death (43). Serum LBP is a stable indicator of serum LPS levels (44). The results showed that the blood LBP level in group PC and group D was higher than that in group B, which presented a U-shaped trend. This indicated that in both the absence of protective effects from *L. salivarius* Li01 and the excessive amount of *L. salivarius* Li01, the mice with ALF and hyperammonemia that were induced by TAA were susceptible to invasion of intestinal bacteria. In addition, LPS from Gram-negative bacteria played a pivotal role that led to deteriorated condition of group PC, which, in combination with brain intoxication caused by high ammonia levels, resulted in a low survival rate in this group. Conditions improved when the mice were pretreated with *L. salivarius* Li01 in groups A and B due to their ability to protect the intestinal barrier, alleviate liver damage, and decrease the plasma and fecal ammonia levels (29, 32). However, when the concentration of *L. salivarius* Li01 continued to increase, the protective effect of the probiotic started to dissipate, and the adverse effects started to emerge. When pretreated with excessive concentrations of *L. salivarius* Li01, the immunocompromised mice, which had a damaged intestinal barrier caused by administration of TAA, were more likely to be afflicted by opportunistic infections. In addition, the excessive concentrations of *L. salivarius* Li01 might cause dysbiosis, where the excessive growth of some bacteria might be responsible for the deterioration in the high-concentration groups, such as group D.

In addition, as CD14 is, like LBP, an intermediate important for the TLR4-MD2 complex to recognize LPS, the relative mRNA expression of CD14 in the liver had a trend similar to that of the blood LBP level, which helped to confirm our results. Blood LBP or LPS levels caused the concentration-related results because the LPS or endotoxins are toxic compounds from bacterial rather than human origin. They could be regarded as external factors, while other parameters, such as ALT, AST, or inflammatory factors, are related to the human body, which was affected by the physical conditions.

In addition, gut microbiota played a role. From the fecal 16S rRNA gene sequencing results, group B was rich in bifidobacterial evolutionary branches. *Bifidobacterium* has been applied as a probiotic for a long time, and a new study demonstrated that many species of *Bifidobacterium* exhibited the ability to improve microbial GABA production, which is known to help in maintaining the physical and psychiatric health of the host (45). The benefit to psychiatric health is particularly important in hyperammonemia, which often leads to psychiatric symptoms. In contrast, group D was rich in *Gammaproteobacteria*, which includes several medically and scientifically important groups of bacteria, such as the families *Enterobacteriaceae*, *Vibrionaceae*, and *Pseudomonadaceae*; many pathogens belong to this class (46). The diversity of these invasive pathogens could explain why group D had more severely damaged intestinal barriers and higher blood LBP levels. Finally, the combination of the above-mentioned adverse factors resulted in inferior survival rate.

The study discovered that a concentration-dependent protective effect of *L. salivarius* Li01 on TAA-induced acute liver injury and hyperammonemia does not exist; contrarily, it exhibits an inverted U-shaped protective effect. The excessive dose of probiotics could generate counterproductive effects. Therefore, the dose should be controlled within a certain range. In addition, when probiotic supplementation is applied to immunocompromised patients, attention should be paid to its risks (47–50).

Finally, there are some limitations to this study. Only four different concentrations of *L. salivarius* Li01 were used in this study, which is not sufficient to obtain accurate concentration range and optimal concentration range of *L. salivarius* Li01. In future studies, more concentrations and administration intervals should be applied. In addition, a better combination of probiotics and a more effective dose regimen to improve endotoxemia of mice in the high-concentration group need to be determined, which could provide more possibilities for the application of *L. salivarius* Li01.

## MATERIALS AND METHODS

### Probiotic strain and culture conditions.

*L. salivarius* Li01 (CGMCC 7045) was isolated and purified from healthy individuals. The bacteria were cultured in MRS liquid medium (Oxoid Ltd., Basingstoke, Hampshire, UK) at 37°C for 24 h in an anaerobic environment. Then, the culture was centrifuged at 3,000 rpm, the supernatant was discarded, and the precipitate was washed twice with sterile normal saline. Finally, the suspension was diluted and concentrated by measuring the absorbance of the suspension at 630 nm (optical density [OD] of 0.6 to 0.8) (16) for four concentration ranges (10^5^ to 10^6^, 10^7^ to 10^8^, 10^9^ to 10^10^, and 10^11^ to 10^12^ CFU/mL, respectively, for the experimental groups A, B, C, and D). For each concentration, the suspensions of *L. salivarius* Li01 were prepared within 30 min before use.

### Animal model and experimental procedure.

Six-week-old male C57BL/6 mice (Ziyuan Ltd., Hangzhou, China) were first housed for 7 days to adapt to the environment (20°C to 22°C, 12-h light and dark cycle) before the experiment. Then, the mice were randomly divided into six groups: NC (*n* = 13), PC (*n* = 17), and groups A, B, C, and D (*n* = 17 in each group). Mice of different groups were housed in different cages to make sure there was no cross-transmission of bacteria.

First, the mice were given 0.3 mL of corresponding liquid (mice in groups NC and PC were given normal saline and those in groups A, B, C, and D were given the corresponding suspensions of *L. salivarius* Li01 mentioned previously; the absolute dosages are 3 × 10^4^ to 3 × 10^5^, 3 × 10^6^ to 3 × 10^7^, 3 × 10^8^ to 3 × 10^9^, and 3 × 10^10^ to 3 × 10^11^ CFU, respectively) by gavage at a fixed time every day for eight consecutive days. Half an hour after the end of gavage on the 8th day, all groups except for group NC were given a single intraperitoneal administration of 300 mg/kg TAA (Sigma-Aldrich, St. Louis, MO, United States), and group NC was injected with the corresponding volume of normal saline (Fig. 1a).

Twenty-four hours after TAA administration, the feces from the mice were collected. After TAA administration, the condition of mice was monitored and recorded every 30 min, and then the survival curve was drawn. After 48 h of TAA administration, the mice that survived were collectively anesthetized and sacrificed, and the blood, livers, intestines, brain tissues, and intestinal contents were collected and stored (−80°C) for subsequent experiments.

### Biochemical analysis and ammonia test of intestinal contents.

The blood that was collected from the mice was contained in a procoagulation tube (BD Vacutainer, Franklin Lakes, NJ, USA), and a small portion was contained in an ethylenediamine tetra-acetic acid anticoagulant tube (BD Vacutainer, Franklin Lakes, NJ, USA). After no agitation for 30 min, the blood was centrifuged at 3,000 rpm for 15 min at 4°C, and the serum/plasma was collected and stored at −80°C.

ALT, AST, and ammonia levels in plasma were measured using a dry chemistry analyzer (FUJI DRI-CHEM 7000V, FUJIFILM, Tokyo, Japan), and LBP levels were detected using an LBP enzyme-linked immunosorbent assay kit (Guduo, Shanghai, China). The ammonia levels in intestinal contents were tested by an ammonia assay kit (Abcam, Cambridge, MA, USA), following the manufacturer’s instructions.

### Histopathology, immunohistochemistry analysis, and immunofluorescence staining.

Left liver and colon tissues were collected in an appropriate size immediately after sacrificing and immersed in 4% paraformaldehyde for 24 h at room temperature. After dehydration and fixation, the tissues were embedded in paraffin. Then, after sectioning, the liver tissues were stained with H&E, and the degree of liver injury was scored according to a histological scoring table (51).

In addition, immunohistochemistry analysis with MPO antibody (AbCam, Cambridge, UK) was used to observe the aggregation of neutrophils in liver tissues, and the steps included dewaxing, repairing, sealing, adding the first and second antibodies, DAB staining, hematoxylin restaining, and neutral gum sealing. A semiquantitative scoring for MPO immunohistochemistry staining was performed by QuantCenter (version 2.2.1.23379, 3D Histech, Budapest, Hungary), and the method was based on previous studies (52). The H-score is calculated by adding up all the proportion of inflammatory cells multiplied by the staining reactivity. The score ranges from 0 to 300. A score of <50 is considered negative, 50 to 100 weakly positive (1+), 101 to 200 moderately positive (2+), and 201 to 300 strongly positive (3+).

The colon was stained with ZO-1 antibody (Proteintech, Rosemont, IL, USA) by immunofluorescence methods according to the previous literature (53). Pathological images were collected by P250 FLASH (3D Histech, Budapest, Hungary).

### RNA extraction and real-time quantitative PCR analysis.

Total RNA in the liver and cortex tissues was extracted by Rneasy minikit (Qiagen, Hilden, Germany) according to the manufacturer’s instructions. Then, the RNA was reverse transcribed as cDNA by PrimeScript RT master mix (TaKaRa Biomedicals, Kusatsu, Japan) as the sample template for subsequent real-time quantitative PCR analysis (RT-qPCR) to detect the relative abundance of mRNA in each tissue. Three replicates were set up and β-actin was used as an internal reference for RT-qPCR. The reagent for RT-qPCR was SYBR premix ex *Taq* II (TaKaRa Biomedicals, Kusatsu, Japan), and the instrument was Applied Biosystems VIIA7 RT-PCR system. The primer information is listed in Table S2.

### Western blotting protein quantitative analysis.

Twenty to thirty milligrams of colon tissue was weighed and put into enzyme-free screw cap micro tube (Sarstedt, Nümbrecht, Germany), which was preloaded with RIPA lysis buffer (Beyotime, Shanghai, China), phenylmethanesulfonyl fluoride (Beyotime, Shanghai, China), and glass beads. Then, the tissue was homogenized and centrifuged to extract the total protein, which was later measured by BCA protein assay kit (Beyotime, Shanghai, China). The protein in each sample was adjusted to a same concentration before being separated by sodium salt polyacrylamide gel electrophoresis. After separation, transmembrane was conducted to transfer the protein onto NC membrane. After washing and blocking, a rabbit anti-Occludin antibody (AbCam, Cambridge, UK) was used to react with the target protein and GAPDH was used as the control. Finally, a horseradish peroxidase (HRP)-labeled goat anti-rabbit IgG was used to bind the previous antibody before developing.

The relative Occludin expression was measured and calculated by Image J (Rawak Software Inc., Stuttgart, Germany), the integrated density of each sample was measured three times, and the average was used to calculate the relative expression.

### Transmission electron microscope.

Approximately 0.5 cm of the colon sample was cut into four pieces in the longitudinal direction and then immersed in 2.5% glutaraldehyde solution for 24 h at 4°C. After washing in phosphate-buffered saline (PBS), the samples were fixed with 1% osmic acid and washed again with PBS, underwent dehydration with a graded series of ethanol and absolute acetone, and then were coated with Spurr resin. The samples were sectioned into 70- to 90-nm slices using an ultramicrotome (Leica EM UC7, Wetzlar, Germany) and were later stained with lead citrate solution and 50% ethanol saturated solution of uranyl acetate. Finally, the prepared samples were observed in Hitachi H-7650 (Hitachi, Tokyo, Japan) TEM.

### Analysis of 16S rRNA gene in gut microbiota.

Total bacterial DNA was extracted from feces that were collected after 24 h of TAA administration by QIAamp fast DNA stool minikit (Qiagen, Hilden, Germany), and then the DNA extract was detected by agarose gel electrophoresis to ensure concentration and purity. Then, the genomic DNA was diluted to 0.1 ng/μL as a template. Combined with specific primers with barcode, high-fidelity PCR master mix (Phusion, New England Biolabs, Ipswich, MA, USA), and GC buffer, the 16S rRNA genes of distinct regions (V3–V4) were amplified by PCR. Then, the PCR products were detected by electrophoresis with 2% agarose gel and purified by magnetic beads. Then, the samples were mixed equally according to the concentration of PCR products. When thoroughly mixed, the mixture was detected by electrophoresis with 2% agarose gel. The target strips were retrieved by a gel recovery kit (Qiagen, Hilden, Germany). Then, sequencing libraries were established using TruSeq DNA PCR-free sample preparation kit (Illumina, San Diego, CA, USA) following the manufacturer’s recommendations. The library quality was assessed by Qubit@ 2.0 fluorometer (Thermo Fisher Scientific, Waltham, MA, USA) system and qPCR. Finally, the qualified library was sequenced on the NovaSeq6000 platform (Illumina, San Diego, CA, USA).

Sequencing data analysis, OTUs cluster, and species annotation are introduced in the supplemental material.

### Statistical analysis.

The differences in Kaplan–Meier survival curves were analyzed by log-rank test. The statistical significance between the groups in ALT, AST, LBP, ammonia, mRNA expression, Western blotting quantitative analysis, and relative abundance of gut microbiota was analyzed by analysis of variance, and correction for multiple comparisons was performed by Tukey’s multiple-comparison test. The difference in pathological scores with ordinal variables was analyzed by nonparametric tests, and correction for multiple comparisons was performed by Dunn’s multiple-comparison test. Shannon index and UniFrac distance were calculated by Qiime (version 1.9.1), and alpha diversity index and beta diversity index were analyzed by R software. PCoA was analyzed by WGCNA, stats, and ggplot2 of R software. LEfSe analysis was performed by LEfSe software (54). Anosim analysis was completed by the Anosim function of the R vegan package. SPSS 20.0 (SPSS, Chicago, IL, USA) and GraphPad Prism 8 (GraphPad software, San Diego, CA, USA) were used to complete the statistical analysis and graphical drawings.

### Ethics statement.

All procedures were performed according to the 2011 National Institutes of Health Guide for the Care and Use of Laboratory Animals and were approved by the Animal Care and Use Committee of the First Affiliated Hospital, School of Medicine, Zhejiang University.

### Data availability.

All data generated or analyzed during this study are included in this published article and its additional information files. The 16S rRNA gene sequencing data have been uploaded to the Sequence Read Archive (SRA) database under BioProject number PRJNA756132.
