# Supplementary material for: Dose-Dependent Relationship between Protection of Thioacetamide-Induced Acute Liver Injury and Hyperammonemia and Concentration of Lactobacillus salivarius Li01 in Mice
Source: Microbiol Spectr. 2021 Dec 22;9(3):e01847-21. doi: 10.1128/spectrum.01847-21 (PMC8694139; doi:10.1128/spectrum.01847-21)
Supplement: SUPPLEMENTAL FILE 1 — Supplemental material. Download SPECTRUM01847-21_Supp_1_seq4.pdf, PDF file, 0.1 MB [file spectrum01847-21_supp_1_seq4.pdf]

Supplementary material

Table S1. Analysis of similarities (Anosim)

Groups	R-Value	*p*-Value
NC versus PC	0.3519	0.004**
NC versus A	0.3352	0.033*
NC versus B	0.3796	0.008**
NC versus C	0.2685	0.018*
NC versus D	0.363	0.006**
PC versus A	0.237	0.059
PC versus B	0.3093	0.034*
PC versus C	0.137	0.122
PC versus D	0.4019	0.002**
A versus B	0.1296	0.136
A versus C	0.5759	0.008**
A versus D	0.4333	0.002**
B versus C	0.3537	0.019*
B versus D	0.3722	0.001***
C versus D	0.2519	0.024*

*$p < 0.05$, **$p < 0.01$, ***$p < 0.001$.

Table S2. PCR primer sequences

Name	Pre-primer	Post-primer
IL-1β	GCAACTGTTCCTGAACTCAACT	TCTTTTGGGGTCCGTCAACT
IL-6	CTGCAAGAGACTTCCATCCAG	AGTGGTATAGACAGGTCTGTTGG
TNF-α	AGGCACTCCCCCAAAAGAT	CAGTAGACAGAAGAGCGTGGTG
BDNF	TCATACTTCGGTTGCATGAAGG	AGACCTCTCGAACCTGCCC
CD14	ACTTCTCAGATCCGAAGCCAG	CCGCCGTACAATTCCACAT

Sequencing data analysis, OTU cluster, and species annotation

After sequencing, according to the barcode sequence and PCR primer sequence, each sample data was separated from the overall data. Then, the barcode sequence and primer sequence were cut off, the reads of each sample were merged into raw tags by FLASH (v1.2.7)(1), which were further filtered to obtain high-quality clean tags (2), according to the QIIME(3) quality control process. The tags were compared with a reference database (Silva database) and then the UCHIME algorithm (4) was applied to detect chimera sequences, finally, the effective tags were obtained by removing the chimera sequences (5).

The algorithm of Uparse (6) (v7.0.1001) was used to cluster all effective tags in all samples and sequences with ≥97% identity similarity were assigned to the same OTUs by default. The representative sequence for each OTU was screened for further annotation. For each representative sequence, the Silva Database(7) was used to annotate taxonomic information based on the Mothur algorithm. Multiple sequence alignment was performed by MUSCLE software(8) to study phylogenetic relationships between different OTUs and the differences between the dominant species in different samples or groups. Finally, by taking the sample with the least amount of data as a standard, the data for each sample was normalized, and subsequent analyses of alpha diversity and beta diversity were all based on this normalized data output.

References

1. Magoč T, Salzberg SL. 2011. FLASH: fast length adjustment of short reads to improve genome assemblies. Bioinformatics 27:2957-63.

2. Bokulich NA, Subramanian S, Faith JJ, Gevers D, Gordon JI, Knight R, Mills DA, Caporaso JG. 2013. Quality-filtering vastly improves diversity estimates from Illumina amplicon sequencing. Nat Methods 10:57-9.

3. Caporaso JG, Kuczynski J, Stombaugh J, Bittinger K, Bushman FD, Costello EK, Fierer N, Peña AG, Goodrich JK, Gordon JI, Huttley GA, Kelley ST, Knights D, Koenig JE, Ley RE, Lozupone CA, McDonald D, Muegge BD, Pirrung M, Reeder J, Sevinsky JR, Turnbaugh PJ, Walters WA, Widmann J, Yatsunenko T, Zaneveld J, Knight R. 2010. QIIME allows analysis of high-throughput community sequencing data. Nat Methods 7:335-6.

4. Edgar RC, Haas BJ, Clemente JC, Quince C, Knight R. 2011. UCHIME improves sensitivity and speed of chimera detection. Bioinformatics 27:2194-200.

5. Haas BJ, Gevers D, Earl AM, Feldgarden M, Ward DV, Giannoukos G, Ciulla D, Tabbaa D, Highlander SK, Sodergren E, Methé B, DeSantis TZ, Petrosino JF, Knight R, Birren BW. 2011. Chimeric 16S rRNA sequence formation and detection in Sanger and 454-pyrosequenced PCR amplicons. Genome Res 21:494-504.

6. Edgar RC. 2013. UPARSE: highly accurate OTU sequences from microbial amplicon reads. Nat Methods 10:996-8.

7. Quast C, Pruesse E, Yilmaz P, Gerken J, Schweer T, Yarza P, Peplies J, Glöckner FO. 2013. The SILVA ribosomal RNA gene database project: improved data processing and web-based tools. Nucleic Acids Res 41:D590-6.

8. Edgar RC. 2004. MUSCLE: multiple sequence alignment with high accuracy and high throughput. Nucleic Acids Res 32:1792-7.
